# Assessing the adaptive potential of European beech populations to temperature and precipitation along a steep environmental gradient in the south‐eastern Carpathians

**DOI:** 10.1111/plb.70129

**Published:** 2025-10-24

**Authors:** M. Tost, O. Grigoriadou‐Zormpa, S. Wilhelmi, M. Müller, H. Wildhagen, A. L. Curtu, O. Gailing

**Affiliations:** ^1^ Division of Plant Breeding Methodology, Department of Crop Sciences University of Göttingen Göttingen Germany; ^2^ Department of Forest Genetics and Forest Tree Breeding University of Göttingen Göttingen Germany; ^3^ Center for Integrated Breeding Research (CiBreed) University of Göttingen Göttingen Germany; ^4^ Faculty of Resource Management, HAWK Göttingen Germany; ^5^ Department of Silviculture Transilvania University of Brasov Brașov Romania

**Keywords:** Climate change, environmental association analysis, *Fagus sylvatica*, local adaptation

## Abstract

- It is necessary to assess the adaptive potential of European beech populations to climate change. Environmental association analysis is a powerful tool for identifying gene loci that contribute to local adaptation to environmental pressures.
- Genotypic data were collected from ~100 adult beech trees per stand in five locations in the south‐eastern Romanian Carpathians along an altitudinal gradient associated with precipitation and temperature. In total, 53 environmental variables, e.g., temperature and precipitation, were used to perform environmental association analysis using LFMM (latent factor mixed models) to identify Single Nucleotide Polymorphism (SNP) markers associated with these. In addition, the principal components (PC) from a principal components analysis (PCA) performed on all environmental variables were used to perform an environmental association analysis.
- We identified 446 SNP markers significantly associated with the first PC. These overlapped with the SNP markers significantly associated with most environmental variables. The first PC was correlated with all temperature‐based variables at |*r*| ~0.989 to ~0.997. A high peak on chromosome 2 from ~4.56 to ~16.27 Mb appeared in all results. This region was ~3.47 Mb downstream of a region for local adaptation identified by Lazic *et al.* (2024). In this peak, 273 markers located in the coding region of 22 genes were found.
- The two genes, *polygalacturonase QRT3‐like* and *NRT1/PTR_FAMILY 5.4‐like*, may be involved in local adaptation based on our literature review. *Polygalacturonase QRT3‐like* plays a role in pollen development in *Arabidopsis*. At the corresponding SNP markers, there was a correlation of the minor allele frequency and temperature‐based environmental variables.

## Graphical abstract

This study focuses on the identification of candidate SNPs involved in local adaptation to altitude by environmental association analysis conducted on different natural European beech (*Fagus sylvatica*) populations.

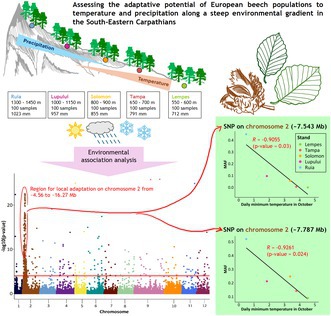

## INTRODUCTION

Many forest tree species are negatively affected by the direct impact of drought stress due to climate change (Hartmann *et al*. 2022; Piedallu *et al*. 2023). Published observations also report that European beech (*Fagus sylvatica* L.) shows increased mortality due to more frequent summer droughts (Martinez del Castillo *et al*. 2022). European beech is distributed across large parts of Europe and can be found from the south of Italy up to the south of Norway (Houston Durrant *et al*. 2016). It is one of the most important forest tree species in Europe, accounting for the highest percentage of broadleaf growing stock on the continent (Houston Durrant *et al*. 2016; FAO 2020; Rukh *et al*. 2023). Martinez del Castillo *et al*. (2022) report that the 1°C increase in temperature from 1955–1985 to 1986–2016 led to reduced beech tree growth at higher altitudes in Central Europe, such as along the Carpathians. Hartmann *et al*. (2022) describe that European beech forests in central Germany are suffering due to increased late frost events in montane regions. Projections assume a decline in the environmental suitability of beech in south and central Germany (Baumbach *et al*. 2019) and southern Europe (Martinez del Castillo *et al*. 2022).

Hence, the importance of studying the adaptive potential of forests to changing climate conditions by evaluating adaptive loci and their association with environmental variables is increasing (Neale & Kremer 2011). Landscape genomics, also known as environmental association analysis (EAA), aims to identify loci involved in local adaptation and associated with environmental variables (Frichot *et al*. 2013; Berg & Coop 2014; Rellstab *et al*. 2015). Many methods are available for EAA which comprise Bayenv (Berg & Coop 2014) and latent factor mixed models (LFMM) (Frichot *et al*. 2013; Frichot & François 2015). Both methods report low rates of false positive observations in comparison to other EAA methods, because they include correction methods for population structure (Frichot *et al*. 2013; de Villemereuil *et al*. 2014). Most EAA methods have difficulties in distinguishing effects of local adaptation from genetic drift and demographic history (Hancock *et al*. 2008). Neutral markers are then used as a null distribution (Hancock *et al*. 2008). So‐called neutral markers comprise intergenic markers which are not in close proximity to genes and are therefore assumed to only capture genetic variation due to genetic drift and demographic history, but not due to adaptation (Hancock *et al*. 2008). Neutral markers are, however, difficult to identify, and intergenic regions do not necessarily only capture genetic variation due to genetic drift and demographic history (Frichot *et al*. 2013). This is even more difficult with sequencing methods like single primer enrichment technology (SPET) that are designed particularly for SNPs within and close to genes (Scaglione *et al*. 2019). LFMM addresses this problem of confounding effects of genetic drift and demographic history by testing the correlation between environmental and genetic variation while estimating the effects of hidden factors, such as residual levels of population structure (Frichot *et al*. 2013). EAA methods return SNP markers that are associated with tested environmental variables (Frichot *et al*. 2013; Rellstab *et al*. 2015). Identified SNPs could help to detect genes or loci involved in adaptation to specific environmental pressures and be compared to other studies. Previous studies in European beech investigated adaptation to different environments with *F*_*ST*_ outlier approaches (Csilléry *et al*. 2014; Krajmerová *et al*. 2017; Cuervo‐Alarcon *et al*. 2018). Some more recent studies used LFMM and other EAA methods but did not work with the chromosome‐level genome assembly of European beech (Postolache *et al*. 2021; Müller *et al*. 2024). The chromosome‐level genome assembly of European beech enabled us to perform a comprehensive analysis of the identified genes. Few studies have used LFMM and other EAA methods and worked with SNP marker datasets based on the chromosome‐level genome assembly of European beech (Lazic *et al*. 2024).

Sampling for EAA studies is often done along environmental gradients (Rellstab *et al*. 2015). For this study, we used 497 individual trees from five European beech stands in the south‐eastern Romanian Carpathians, sampled along an altitudinal gradient ranging from 550 up to 1400 m a.s.l. associated with precipitation and temperature. In total, 53 environmental variables were investigated separately in EAA using LFMM. Additionally, we also performed a principal component analysis (PCA) on all environmental variables. Performing a PCA on environmental variables can help to reduce the dataset and decrease the number of tests (Rellstab *et al*. 2015). However, the interpretation of the principal components (PCs) can be difficult, and true positives may be missed, leading to misclassifications (Rellstab *et al*. 2015). Hence, it is recommended to only run EAA on environmental PCs when they can be interpreted (Rellstab *et al*. 2015).

The climate conditions in summer of the south‐eastern Romanian Carpathians may be similar to the future climate conditions in mountainous regions of Germany (Baumbach *et al*. 2019; Martinez del Castillo *et al*. 2022). Patterns of fine‐scale local adaptation to different environmental pressures are described along a steep environmental gradient in these populations of European beech. Furthermore, environmental variables involved in local adaptation are identified. Results from this study can help in the adaptive management of European and German beech forests, but there is still a remaining uncertainty regarding future climate conditions (Jandl *et al*. 2019).

## MATERIAL AND METHODS

### Data sampling and stand descriptions

The used dataset consists of 497 individual trees collected from five beech stands in the south‐eastern Romanian Carpathians along an altitudinal gradient associated with precipitation and temperature. The beech stands are referred to as Lempes, Tampa, Solomon, Lupului and Ruia. In each stand, approximately 100 trees were sampled in August 2021 for DNA analyses. Lempes was the lowest elevation site at 550–600 m, followed by Tampa at 650–700 m, Solomon at 800–900 m and Lupului at 1000–1150 m. Ruia was the highest location at 1300–1450 m. The diameter at breast height (DBH) in Ruia averaged 35.7 cm, in Lupului 52.5 cm, in Solomon 29.5 cm, in Tampa 41.3 cm, and in Lempes 47.5 cm. The distribution of DBH measurements across the different stands is available in Fig. S1. For further details see Grigoriadou‐Zormpa *et al*. (2025), in which also fine‐scale spatial genetic structure of the stands is reported.

### Genotypic data

Individual trees were sequenced using single primer enrichment technology (SPET) (Scaglione *et al*. 2019). This sequencing method targets SNPs located within and close to genes and flanking regions of ±2 kb (Scaglione *et al*. 2019). The target regions were determined by previous whole‐genome sequencing of a subset of 96 individual trees by IGATech (IGA Technology Services, Udine, Italy, n.d). Reads were aligned to the *Fagus sylvatica* L. reference genome v. 2 (Mishra *et al*. 2021) using BWA‐MEM v. 0.7.17 (Li & Durbin 2009). Only aligned reads with a mapping quality ≥10 were kept, and duplicated reads were removed. Variant calling was done along with filtering for minimum read number of an allele in a sample using Freebayes v. 1.3.6 (Garrison & Marth 2012; Grigoriadou‐Zormpa *et al*. 2025). Normalization and filtering for raw read depth were performed using bcftools (Danecek *et al*. 2011; Grigoriadou‐Zormpa *et al*. 2025). After this, a total of 838,522 SNP markers were kept, which were used in the downstream analysis.

### Environmental data

In total, 53 environmental variables were used in this analysis. The environmental variables comprise recorded weather data from 1981 until 2010. Environmental data were downloaded from the climatology database CHELSA v. 2.1 (Karger *et al*. 2017; Karger, Schmatz, *et al*. 2020; Karger & Zimmermann 2018; Beck *et al*. 2020; Brun *et al*. 2022). The environmental variables comprised elevation in m above sea level, frost change frequency (fcf), and precipitation and temperature variables (Karger *et al*. 2017; Karger & Zimmermann 2018; Beck *et al*. 2020; Karger, Dabaghchian, *et al*. 2020; Karger, Schmatz, *et al*. 2020; Brun *et al*. 2022). All these variables were available for groups of individual trees, because the coordinates of the trees were recorded and the climatology database CHELSA v. 2.1 has a very high resolution (30 arc sec, ~1 km).

Additionally, the Ellenberg‐Quotient was calculated as $Q_{E}=\frac{{\text{Temperature in}}^{{}^{\circ}}C\;\left(\text{July}\right)*1000}{\text{Precipitation in}\;\mathrm{mm}\;\left(\text{mean}\right)}$ (Ellenberg 2009). Precipitation was modelled as monthly precipitation from January to December, mean annual precipitation and the precipitation accumulated over the growing season (gsp) (Karger *et al*. 2017; Karger & Zimmermann 2018; Beck *et al*. 2020; Karger, Dabaghchian, *et al*. 2020; Karger, Schmatz, *et al*. 2020; Brun *et al*. 2022). The temperature was measured as mean, minimum and maximum daily air temperature from January to December (Karger *et al*. 2017; Karger & Zimmermann 2018; Beck *et al*. 2020; Karger, Dabaghchian, *et al*. 2020; Karger, Schmatz, *et al*. 2020; Brun *et al*. 2022). The distribution of environmental variables across stands is shown in Fig. S2.

We examined the correlations of the environmental variables with each other, which are shown in Fig. S3. Correlations were analysed using R v. 4.3.1 (R Core Team 2024). A principal component analysis (PCA) was performed on all 53 environmental variables using the *princomp ()* R function in the R v. 4.3.1 (R Core Team 2024). The first 14 principal components (PCs) with the largest eigenvalues were also investigated using LFMM. Fig. S4 shows the two first PCs of the PCA on the environmental variables and the eigenvalues of all PCs.

### Environmental association analysis (EAA)

**Fig. 1:**
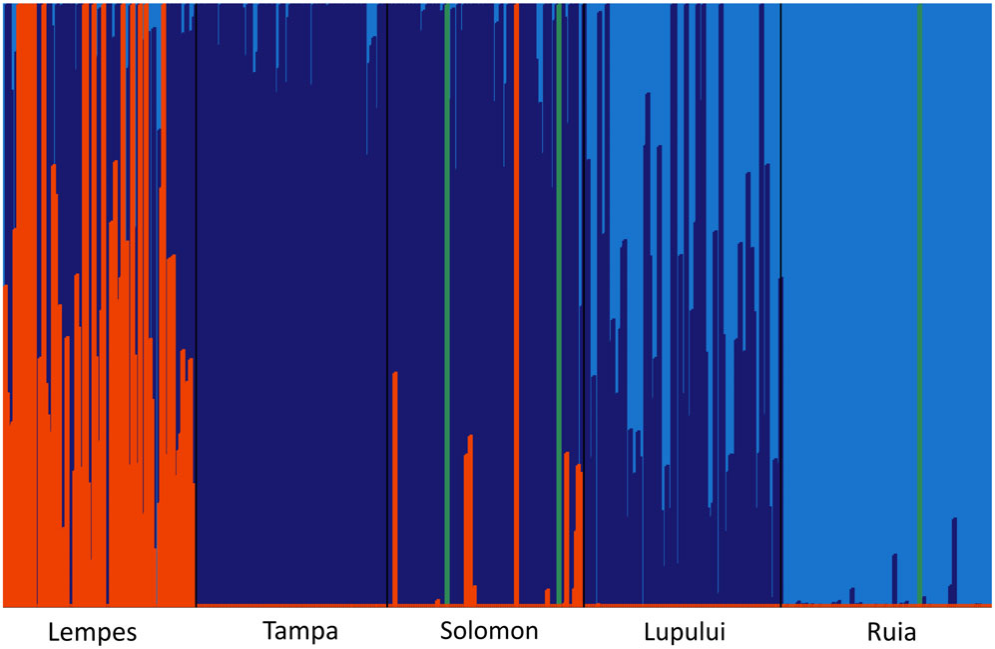
Population structure based on fastStructure (Raj *et al*. 2014) of the analysed five beech stands Lempes, Tampa, Solomon, Lupului, and Ruia. The different colours represent the identified clusters based on the population structure analysis.

To test for associations between SNPs and environmental gradients, we used the latent factor mixed models (LFMM) (Frichot *et al*. 2013) implemented in the LEA (Landscape and Ecological Association Studies) R package (Frichot & François 2015; R Core Team 2024). This LFMM model is an improved version of the LFMM algorithm from Frichot *et al*. (2013), which implements LFMM based on a Bayesian bootstrap approach (Frichot & François 2015). LFMM detects correlations between environmental variables and genetic variation while considering random effects related to population structure (Frichot *et al*. 2013). LFMM estimates confounders, also referred to as latent factors (Frichot *et al*. 2013; Frichot & François 2015). Latent factors are then included in a statistical model for testing associations between genotypes and the environmental variable (Frichot *et al*. 2013; Frichot & François 2015). To run LFMM, missing data had to be imputed (Frichot *et al*. 2013; Frichot & François 2015), after removal of markers with a missingness >0.05 (see Fig. S5). Imputation was then done with the *impute()* function from the LEA R package (Frichot & François 2015; R Core Team 2024). LFMM tested all 53 environmental variables and 14 environmental PCs separately (Frichot *et al*. 2013; Frichot & François 2015). The number of latent factors was set to three based on the results of the population structure analysis (see Fig. 1 and below). LFMM results were then Benjamini‐Hochberg (BH) corrected (Benjamini & Hochberg 1995). BH correction was conducted with the FDRestimation R package (Murray & Blume 2021).

LFMM aims to account for latent structure, but signals of demographic history may still remain and are difficult to disentangle from the signals of adaptation (Frichot *et al*. 2013; Frichot & François 2015). To visualize these spurious remaining signals and to determine if the significance thresholds are appropriate, the number of false‐positive observations exceeding these thresholds was determined by permutation tests. For these permutation tests, the first environmental PC was used. The permutation was performed while maintaining the population or stand structure, with the order of the stands randomized. The stands were randomized by changing the order along the altitudinal gradient to remove association with the altitudinal gradient. This randomization scheme is shown in Table S6. Additionally, the order of individuals within the stands was randomized using the *sample()* R function in R v. 4.4.0 (R Core Team 2024). A total of ten replication schemes with ten replications each were carried out. The results of the randomizations are shown in Table S6. For the significance thresholds based on the BH correction, an average of ~39 false‐positive results were observed for the ten different permutations with ten replications (Table S6).

### Assessing population stratification and assesment of confounders

To infer population structure and to identify the number of confounders in the dataset, the fastStructure algorithm was used (Raj *et al*. 2014). FastStructure uses variational Bayesian inference methods to determine the underlying ancestry proportions, similar to Structure (Pritchard *et al*. 2000), but faster and with a more flexible priority distribution (Raj *et al*. 2014). FastStructure achieves similar accuracies comparable to ADMIXTURE (Raj *et al*. 2014). FastStructure was run in python v. 3.11.6 (Van Rossum & Drake 2009). The results were analysed using R v. 4.3.1 (R Core Team 2024). Results of the population structure analysis are shown in Fig. 1. There is a clear, albeit slight, differentiation between the stands showing different proportions of three main genetic clusters (Fig. 1). These clusters were included as K‐value in the LFMM analysis. Based on these, LFMM aims to account for latent structure, but some signals of demographic history may still contribute (Frichot *et al*. 2013; Frichot & François 2015).

Additionally, we conducted an analysis of molecular variance (AMOVA) to assess the variation among and within stands with the poppr R package (Kamvar *et al*. 2015). According to the AMOVA, the variation among stands was 3.11% and between samples within stands was 96.89% (see Table S7). The pairwise *F*_*ST*_ matrix is shown in Table S8. It was calculated based on the StAMPP R package (Pembleton *et al*. 2013).

## RESULTS

The first environmental principal component (PC) explained 95.04% of the total variance, and the first two PCs accounted together for ~99.5% (see Fig. S4). We found that additional LFMM analysis on subsequent environmental PCs did not yield additional significant associations (see Fig. S9). The first environmental PC was correlated at |*r*| ~0.989 to ~0.997 with all temperature‐based variables and elevation and at |*r*| ~0.950 to ~0.945 with all precipitation‐based variables and EQ. The correlation coefficients with frost frequency change (fcf) and precipitation accumulated over the growing season (gsp) were |*r*| ~0.813 and ~ 0.692.

**Fig. 2:**
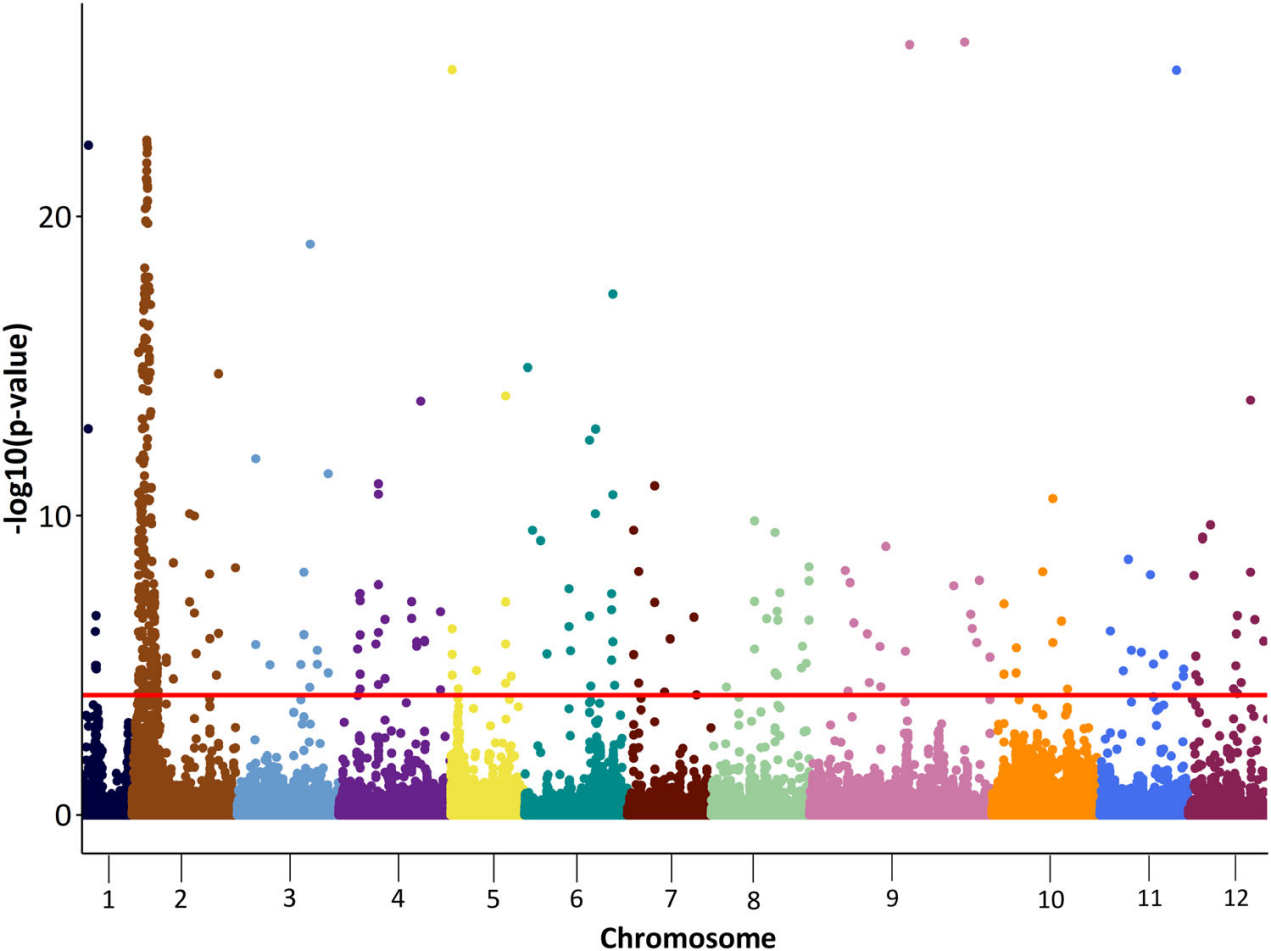
Manhattan plot based on LFMM result for environmental PC1 with Benjamini‐Hochberg (BH) corrected *P*‐values on the –log_10_ scale. The red horizontal line indicates the significance thresholds with *P* ≤ 0.0001.

Figure 2 shows results of the LFMM analysis of the first environmental PC with Benjamini‐Hochberg (BH) corrected *P*‐values on the –log_10_ scale. Environmental PC1 was significantly associated with 446 markers, of which 288 are located on chromosome 2 (Fig. 2). A high peak region appears on chromosome 2 from ~4.56 to ~16.27 Mb, in which 273 markers are located. The LFMM analysis of the individual environmental variables shows a similar pattern except for gsp (see Fig. S10).

**Fig. 3:**
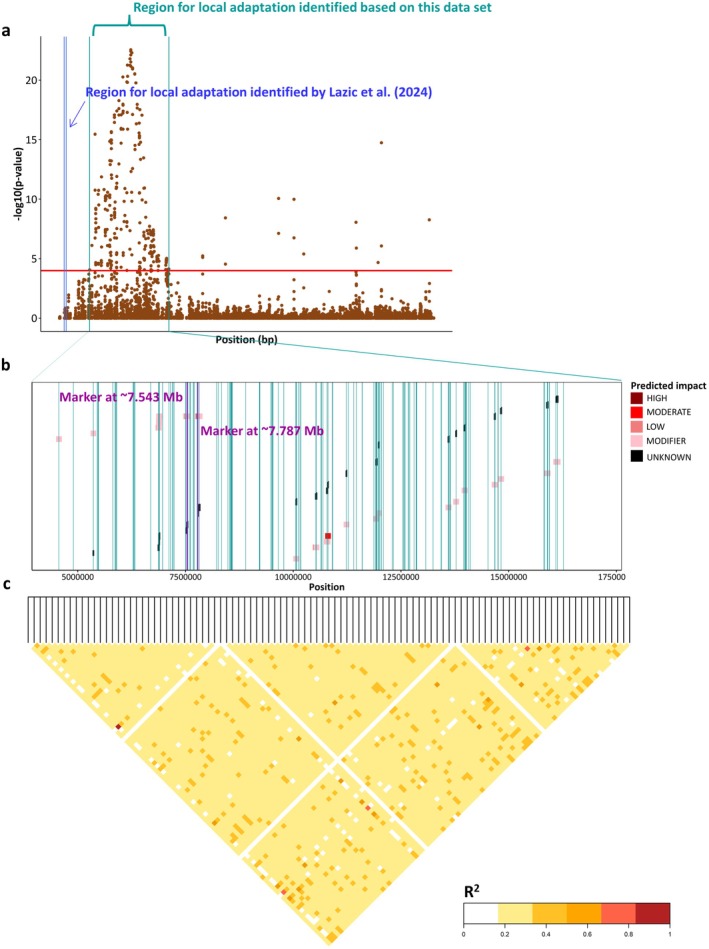
High peak region on chromosome 2 from ~4.56 to ~16.27 Mb with (a) Benjamini‐Hochberg (BH) corrected *P*‐values on the –log_10_ scale (red horizontal line indicates the significance threshold with *P* ≤ 0.0001), the blue vertical line shows the region for local adaptation observed in Lazic *et al*. (2024), (b) the coding regions of underlying genes and significant markers (green vertical lines), and (c) the LD heat map in this region for Solomon.

Figure 3a shows the LFMM results with Benjamini‐Hochberg (BH) corrected *P*‐values on the –log_10_ scale only for chromosome 2. The pairwise linkage disequilibrium (LD) heatmap was calculated for the high peak region on chromosome 2 from ~4.56 to ~16.27 Mb (Fig. 3c). The blue vertical line shows the region for local adaptation observed in the study of Lazic *et al*. (2024) from 0.79 Mb to 1.09 Mb on chromosome 2. The coding regions of all gene variants observed across this region are shown in Fig. 3b. The pairwise LD was 0.2586 in Ruia, 0.2503 in Lupului, 0.2687 in Solomon, 0.2620 in Tampa, and 0.2668 in Lempes (Fig. 3c).

Table 1 shows all significant SNP associations on chromosome 2 within the high peak region. In total, we observed 237 SNP markers located between ~4.56 Mb and ~ 16.27 Mb. Some markers were located within coding regions of 22 different genes, many markers were located within the coding region of the same gene, and some markers were outside of the coding regions of genes (Fig. 3). In total, we found 10 described genes based on our literature review (Table 1).

**Table 1:** List of candidate SNPs with gene annotations associated with environmental PC1 with Bonferroni‐Hochberg corrected *P*‐values at a significance level of *P* ≤ 0.0001 and their annotated gene variants, the underlying genes with their locations, functions and predicted impact with HIGH, MOD (moderate) and LOW based on SnpEff (Cingolani *et al.* 2012).

Chr	marker pos	gene variant			underlying gene	gene description
2	4560315	Bhaga_2.g515	1 missense variant	MOD	XP_023914361.1	NA
	5362037	Bhaga_2.g604	Stop gain variant	HIGH	PSS09972.1	NA
	5362045		2 missense variants	MOD		
	5362069			MOD		
	6876799	Bhaga_2.g787	1 missense variant	MOD	RWR85963.1	NA
	6886424	Bhaga_2.g788	2 missense variants	MOD	XP_023921788.1	NA
	6886446			MOD		
	6890020	Bhaga_2.g789	1 missense variant	MOD	KAB1227540.1	NA
	7513043	Bhaga_2.g853	1 missense variant	MOD	RVW44103.1	NA
	7542764	Bhaga_2.g857	1 missense variant	MOD	**XP_030966603.1**	**polygalacturonase QRT3‐like:** responsible for degrading the pollen mother cell wall during microspore development (Rhee *et al*. 2003, National Center for Biotechnology Information (NCBI) 2025)
	7787374	Bhaga_2.g883	1 missense variant	MOD	**XP_030961693.1**	**protein NRT1/ PTR FAMILY 5.4‐like:** functions as a transporter of indole‐3‐butyric acid (IBA), a precursor of the major endogenous auxin indole‐3‐acetic acid (IAA) (National Center for Biotechnology Information (NCBI) 2025)
	7820389	Bhaga_2.g885	1 missense variant	MOD	XP_023871679.1	
	10071121	Bhaga_2.g1122	2 missense variants	MOD	KAE8713369.1	uncharacterized LOC120113939
	10071153			MOD		
	10526069	Bhaga_2.g1166	2 missense variants	MOD	XP_030967199.1	transcription elongation factor SPT6 homologue
	10533078			MOD		
	10784123	Bhaga_2.g1189	Variant hits 5′UTR region with premature start codon gain variant	LOW	XP_023872570.1	NA
	10805547	Bhaga_2.g1191	2 missense variants	MOD	PON69882.1	NA
	10805606			MOD		
	11233581	Bhaga_2.g1238	Variant hits 5′UTR region with premature start codon gain variant	LOW	NA	NA
	11928940	Bhaga_2.g1303	1 missense variant	MOD	XP_023875148.1	NAD‐dependent malic enzyme 62 kDa isoform, mitochondrial
	11988800	Bhaga_2.g1308	1 missense variant	MOD	XP_012833939.1	ras‐related protein RABH1b
	13600231	Bhaga_2.g1485	1 missense variant	MOD	RVX03908.1	NA
	13781237	Bhaga_2.g1505	1 missense variant	MOD	XP_023915086.1	NA
	13976512	Bhaga_2.g1525	1 missense variant	MOD	XP_023880346.1	NA
	14682297	Bhaga_2.g1609	1 missense variant	MOD	XP_030966443.1	spindle and kinetochore‐associated protein 1 homologue
	14823027	Bhaga_2.g1623	1 missense variant	MOD	XP_030961900.1	protein IWS1 homologue
	15894816	Bhaga_2.g1733	1 missense variant	MOD	XP_030963279.1	protein transport protein SEC16B homologue
	16129816	Bhaga_2.g1756	1 missense variant	MOD	XP_030965516.1	CCR4‐NOT transcription complex subunit 1

Candidate genes that may be involved in local adaptation are highlighted in bold.

**Fig. 4:**
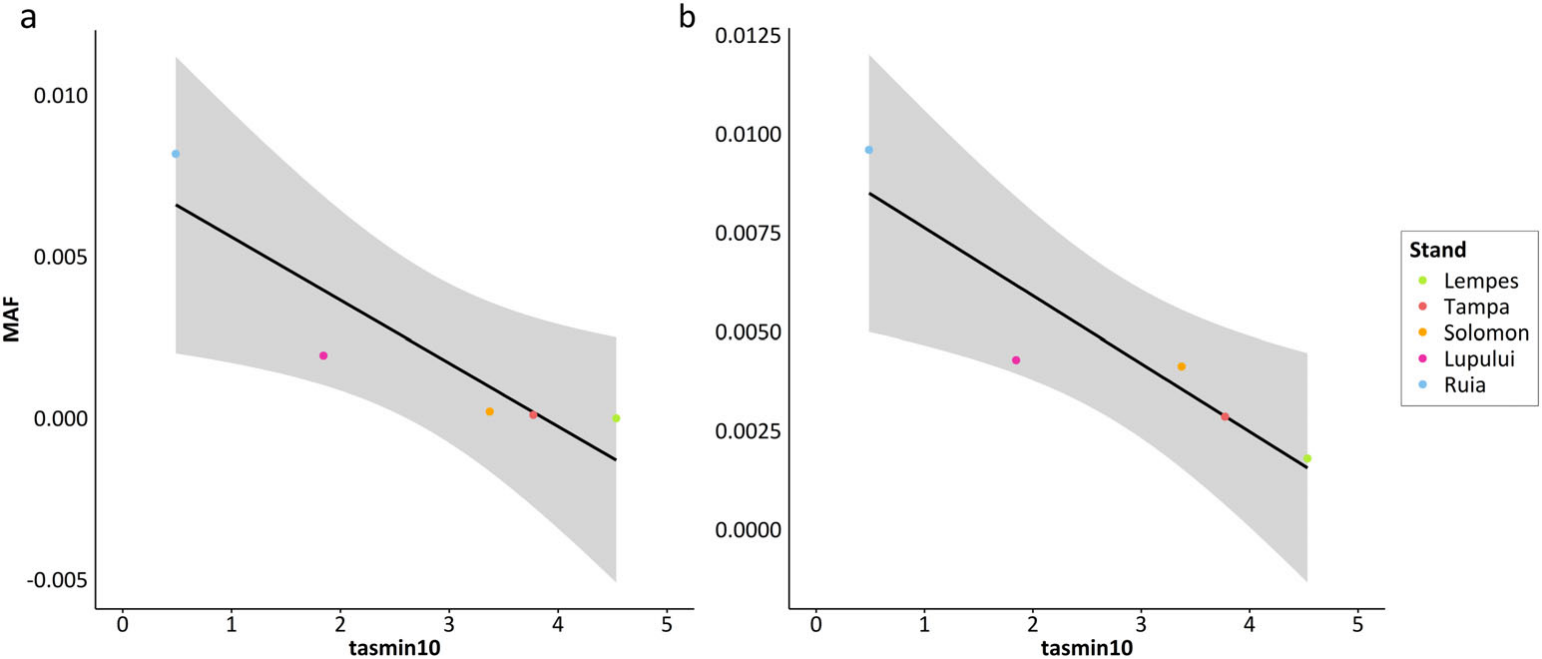
(a) Correlation between minimum daily temperature in October (tasmin10) and the minor allele frequency (MAF) of the significant marker (*P* ≤ 0.0001) on chromosome 2 at ~7.543 Mb with a correlation coefficient of −0.9055 (*P*‐value = 0.03). This marker was annotated with gene variant *Bhaga_2.g857*, with the underlying gene *polygalacturonase QRT3‐like*. (b) Correlation between minimum daily temperature in October and the minor allele frequency (MAF) of the significant marker (*P* ≤ 0.0001) on chromosome 2 at ~7.787 Mb with a correlation coefficient of −0.9261 (*P*‐value = 0.024). This marker was annotated with gene variant *Bhaga_2.g883*, with the underlying gene protein *NRT1/ PTR FAMILY 5.4‐like*.

For the markers on chromosome 2 at ~7.543 Mb and at ~7.787 Mb, we found two interesting underlying candidate genes that may be involved in local adaptation. At both markers, the minor allele frequency (MAF) decreases with increasing minimum daily temperature in October following the altitudinal gradient (Fig. 4). For minimum daily temperature in October, we observed the highest absolute correlation coefficients of |*r|* ~ 0.9055 (*P*‐value = 0.03) and ~ 0.9261 (*P*‐value = 0.024). Significant correlation coefficients of the MAF and the other environmental variables are shown in Fig. S11. For all temperature‐based variables, we observed the same trend (see Fig. S11, Table S12). For all precipitation‐based variables, we observed the opposite trend (see Fig. S11, Table S12). The correlation coefficients of the MAF and precipitation‐based variables, EQ and fcf were not significant. The significant correlation coefficients ranged between |*r*| ~0.8786 and ~ 0.9055 for the marker on chromosome 2 at ~7.543 Mb. For the marker on chromosome 2 at ~7.787 Mb, the significant correlation coefficients ranged between |*r*| ~0.9090 and 0.9261.

**Fig. 5:**
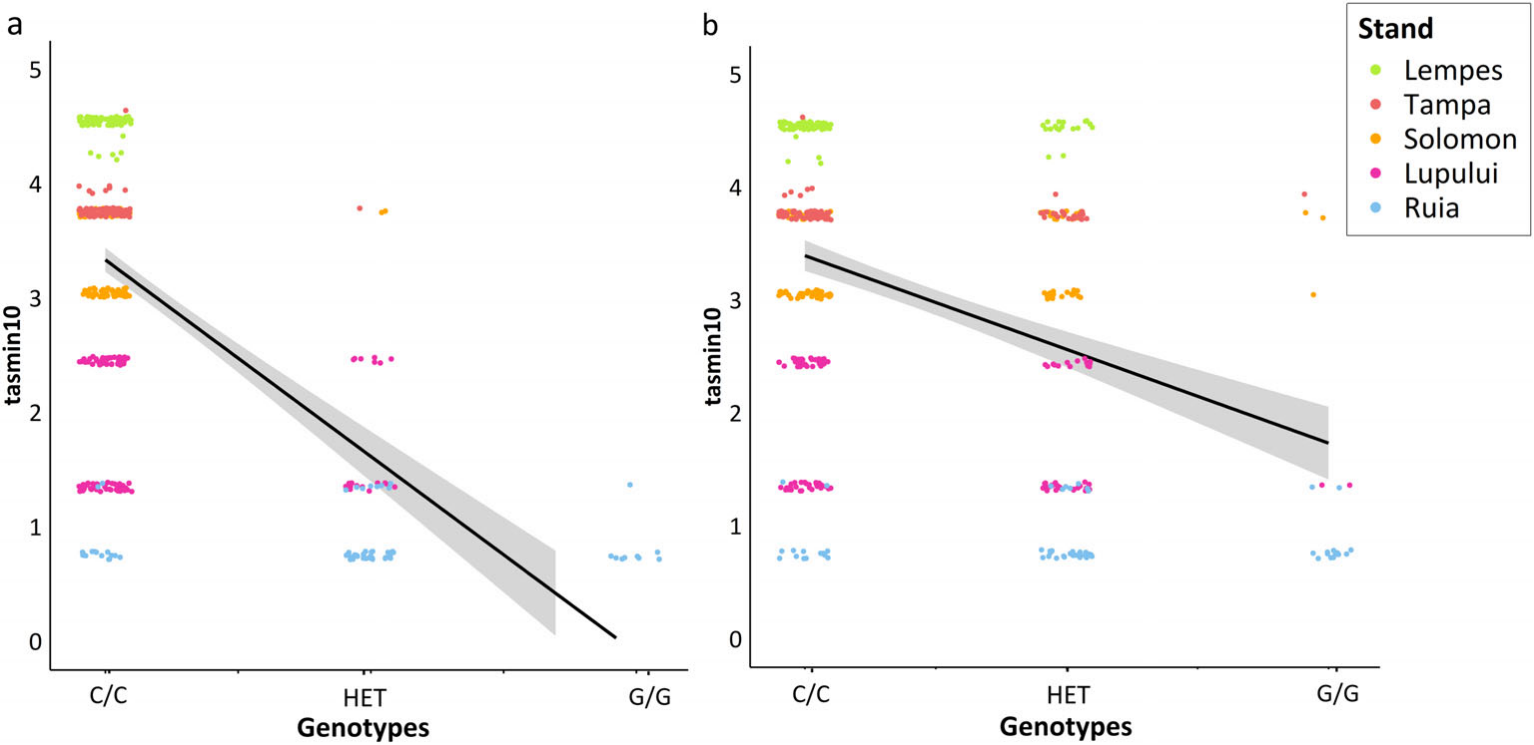
Correlation between minimum daily temperature in October (tasmin10) and observed genotypes at the significant SNP markers on chromosome 2 at ~7.543 Mb (*Bhaga_2.g857*, with the underlying gene *polygalacturonase QRT3‐like*) (a) and 7.787 Mb (*Bhaga_2.g887*, with the underlying gene protein *NRT1/ PTR FAMILY 5.4‐like*) (b). The coefficients of determination R^2^ were 0.3468 (*P* ~ 0) and 0.1646 (*P* ~ 0).

For all other markers on chromosome 2, from ~4.56 to ~16.27 Mb, we observed the same trend (see Fig. S13). In all cases, the minor allele frequency (MAF) is correlated with minimum daily temperature in October (see Fig. S13). Additionally, a linear regression of the different genotypes observed at the SNP markers on chromosome 2 at ~7.543 Mb (*Bhaga_2.g857*, with the underlying gene *polygalacturonase QRT3‐like*) and ~ 7.787 Mb (*Bhaga_2.g883*, with the underlying gene protein *NRT1/ PTR FAMILY 5.4‐like*) and minimum daily temperature in October is shown in Fig. 5. The regression coefficients for SNP markers on chromosome 2 at ~7.543 Mb and at ~7.787 Mb are 0.3468 and 0.1646, respectively (Fig. 5).

## DISCUSSION

### The EAA results strongly correlate with temperature‐based variables

This environmental association analysis aims to assess the adaptive potential to different environmental pressures along a steep environmental gradient in populations of European beech from five locations in the south‐eastern Romanian Carpathians. The altitudinal gradient is associated with changes in precipitation and temperature (see Fig. S2). In total, 53 environmental variables and the principal components calculated based on these were used to perform environmental association analysis using LFMM (latent factor mixed models). We identified 446 SNP markers significantly associated with the first principal component (PC 1). These were overlapping with the SNP markers significantly associated with all environmental variables except precipitation accumulated during the growing season. The first PC was correlated with all temperature‐based variables and elevation at |*r*| ~0.989 to ~0.997 and with all precipitation‐based and Ellenberg‐Quotient variables at |*r*| ~0.945 to 0.950, except precipitation accumulated during the growing season. The 273 markers on chromosome 2 from ~4.56 to ~16.27 Mb, are overlapping or close to 22 genes. In total, we found 10 genes with gene descriptions available based on our literature review (Table 1). Most of these genes are involved in gene regulation processes and enzyme catalyzation, and their impact is difficult to deduce (see Table S14). For some genes, functional studies are only available for homologues in rather distantly related species, such as humans (Table S14).

For two gene variants, we found annotated genes potentially involved in local adaptation to specific environmental conditions and both genes have been studied in plants. These gene variants were *Bhaga_2.g857* and *Bhaga_2.g883*. A high peak region on chromosome 2 from ~4.56 to ~16.27 Mb appeared in all results. This region was ~3.47 Mb downstream from a high‐confidence region for local adaptation identified by Lazic *et al*. (2024) on the same chromosome ~0.79 Mb to 1.09 Mb.

### EEA peak region on chromosome 2 locates close to a previously reported high‐confidence region for local adaptation

Lazic *et al*. (2024) found the gene variant *Bhaga_2g.94*, which was annotated with the gene *Callose synthase 1,* as candidate gene for local adaptation. It was assumed that *Callose synthase 1* could be involved in winter dormancy and spring bud burst (Lazic *et al*. 2024). *Callose synthase 1* is described as involved in callose deposition during microspore development. Microspores are surrounded by a callose wall, this wall is then degraded, and microspores are released (Tylewicz *et al*. 2018; Chu *et al*. 2024). We did not observe any significant SNP markers overlapping or in linkage disequilibrium (LD) with the high‐confidence region from Lazic *et al*. (2024). However, we found a candidate gene based on our literature review, with very similar function to *Callose synthase 1* which was annotated for a marker on chromosome 2 at ~7.543 Mb. This candidate gene is *polygalacturonase QRT3‐like* (*Bhaga_2.g857*). The gene *polygalacturonase QRT3‐like* plays a crucial role in pollen development in *Arabidopsis thaliana* L. (Rhee *et al*. 2003) and *Brassica rapa* L. (Chu *et al*. 2024). The gene function of *polygalacturonase QRT3‐like* is similar to *Callose synthase 1* (Rhee *et al*. 2003; Tylewicz *et al*. 2018; Chu *et al*. 2024) and they are often studied together (Rhee *et al*. 2003; Chu *et al*. 2024). For further studies we recommend not only to consider the markers from this study, but also the region identified by Lazic *et al*. (2024). Our results provide statistical support for local adaptation on a small geographic scale in the face of gene flow along an environmental gradient. Lazic *et al*. (2024) studied local adaptation in 98 populations distributed from Southern Europe to Central and Eastern Europe.

### The candidate gene *polygalacturonase QRT3‐like*

The gene variant *Bhaga_2.g857* corresponds to the underlying gene *polygalacturonase QRT3‐like* (NCBI 2025). We found one SNP on chromosome 2 at ~7.543 Mb located within the coding region of *Bhaga_2.g857* (*polygalacturonase QRT3‐like*) (Fig. S15). At this position of the coding region, a missense variant with moderate impact is located causing a codon change, which leads to a change in the polar bonds of the amino acid (Table S16). The minor allele frequency at this marker decreased with increasing minimum daily temperature in October. The same significant correlation trend was observed for many temperature‐based variables (see Fig. S11). The correlation with precipitation‐based variables or other environmental variables like EQ, frost frequency change (fcf), and elevation was not significant. Our results suggest that this gene may be involved in local adaptation to temperature. But also other environmental variables such as elevation, precipitation‐based variables and EQ may have contributed slightly to the association with the first environmental PC. Hence, the first PC not only represents temperature‐based variables but also includes elevation and precipitation‐based variables.

Furthermore, a splice region variant of the coding region of this gene with low impact is located 70 bp upstream from the highly significant SNP marker (Fig. S15). The marker at the position of the splice region variant and the other missense variants are contained as markers in the dataset but are not significant. High LD is observed across the coding region of *Bhaga_2.g857* (*polygalacturonase QRT3‐like*), but the LD is incomplete across the entire coding region of the gene (Fig. S15). It is possible that these markers do not exceed the significance threshold because of insufficient power of the study (Sun *et al*. 2022). If very strict significance thresholds are chosen, only the coding region variants with the highest impact and at higher allele frequency in the population will be detected (Sun *et al*. 2022). In addition to the missense variants already mentioned, there are seven further missense variants between 21 bp upstream and 142 bp downstream of the marker (see Fig. S15).

The high‐confidence region on chromosome 2 from ~0.79 to 1.09 Mb from Lazic *et al*. (2024) was identified using three different methods for environmental association analysis (EAA). These three methods comprised LFMM from the LEA package (Frichot *et al*. 2013; Frichot & François 2015), Baypass (Gautier 2015) and WZA (Capblancq & Forester 2021). Additionally, Lazic *et al*. (2024) also measured the relative expression of *Callose synthase 1* in winter buds of eight beech trees. They observed that the relative expression level of *Callose synthase 1* was increased, but not differentially expressed, in the individuals carrying the alternative allele at the SNP marker identified as top LFMM hit on chromosome 2 at ~0.898 Mb (Lazic *et al*. 2024). The individual trees carrying the homozygous genotype of the alternative allele at this SNP marker showed, in a common garden experiment, less days until bud burst (Lazic *et al*. 2024). Furthermore, a clear pattern of geographic distribution across the sampling area from Southern Europe to Central and Eastern Europe was observed (Lazic *et al*. 2024). The alternative allele increased towards Central and Eastern Europe; in this region, the temperature of the coldest month was much lower (Lazic *et al*. 2024).

The small geographic scale of this single altitudinal profile analysed in the present study offers a strong basis for detecting local adaptation but may also limit the applicability of the results to other regions within the broader range of *F. sylvatica* (Rellstab *et al*. 2015; Houston Durrant *et al*. 2016). In contrast, the study of Lazic *et al*. (2024) captures large parts of the distribution area of European beech. To compare patterns of local adaptation at small and range‐wide geographic scales, in future studies we will assess our candidate SNPs also at a larger geographic scale across the species distribution range. Furthermore, replication of gradients is important to corroborate candidate SNPs for local adaptation (Rellstab *et al*. 2015). The use of replicates can also reduce confounding effects due to undetected population structure or demographic history (Rellstab *et al*. 2015).

### The candidate gene *NRT1/PTR_FAMILY 5.4‐like*

For the gene variant *Bhaga_2.g883*, the annotated gene *NRT1/PTR_FAMILY 5.4‐like* was found (National Center for Biotechnology Information (NCBI) 2025). The gene *NRT1/PTR_FAMILY 5.4‐like* belongs to the NRT1/PTR family. This family was initially described as nitrate transporters, but recently their involvement in the transport of phytohormones like auxin was discovered (Chiba *et al*. 2015; Watanabe *et al*. 2020). Many different members of this gene family have been studied (Bai *et al*. 2013; Chiba *et al*. 2015; Watanabe *et al*. 2020), but *NRT1/PTR_FAMILY 5.4‐like* has not been studied in detail to our knowledge. But we can assume that this particular gene may also be involved in the transport of phytohormones like auxin. The gene *NRT1/PTR_FAMILY* serves as a transporter for indole‐3‐butyric acid (IBA), which is a precursor of indole‐acetic acid (IAA) (Watanabe *et al*. 2020). IAA is the major form of naturally available auxin (Chiba *et al*. 2015; Watanabe *et al*. 2020). At the position of the marker on chromosome 2 at ~7.787 Mb, a missense variant with moderate impact was found in the coding region of *Bhaga_2.g883* (*NRT1/PTR_FAMILY 5.4‐like*) (Fig. S17). This missense variant causes a codon change which replaces a hydrophilic with a hydrophobic amino acid (Table S16). This change can increase the stability of the proteins (van Dijk *et al*. 2015) involved in auxin transport in the Ruia stand. Additionally, one splice region variant of the coding region of *Bhaga_2.g883* (*NRT1/PTR_FAMILY 5.4‐like*) was located 83 bp upstream from this significant marker (Fig. S17). This splice region variant was not recognized as significant in our analysis, which could also be related to the fact that it has less influence on the coding region. LD varies strongly across the gene coding region of *Bhaga_2.g883* (*NRT1/PTR_FAMILY 5.4‐like*) (Fig. S17). It seems like the splice region variant is not in strong LD with the marker and the rest of the coding region of this gene (Fig. S17). The missense variant in the coding region is, however in almost complete LD (~1) with the marker (Fig. S17).

Figure 4b shows that the MAF at the marker on chromosome 2 at ~7.787 Mb decreased with increasing minimum daily temperature in October. The same trend was observed for all temperature‐based variables and for elevation, with a significance level of *P* ≤ 0.05. The correlation coefficient for MAF at this marker and precipitation in October was ~0.81 (*P*‐value = 0.096), the correlation coefficients for the other precipitation‐based variables except gsp were similar. Auxin activity is decreased during drought stress (Popko *et al*. 2010). A decrease in free auxin in the cambial zone of poplar was observed in acclimation to drought stress caused by osmotic stress related to changes in water potential in the environment (Popko *et al*. 2010). In general, high auxin activity is correlated with high growth rates in trees (Popko *et al*. 2010). Since this particular gene was not studied, it is difficult to draw direct conclusions about the exact involvement in local adaptation, but we would assume that auxin is decreased in the shoot apical meristems of the stands with high drought stress. The allele frequency at the SNP candidate marker on chromosome 2 at ~7.787 Mb is decreased in the stands Lempes, Tampa, and Solomon, with lower precipitation and higher temperatures (see Fig. 4b). Only in Ruia is the allele frequency at the SNP candidate increased (see Fig. 4b).

Both gene variants may be involved in local adaptation. This is hypothetical and only based on our literature review. In order to substantiate these assumptions, expression levels of the genes should be measured under contrasting environmental conditions, or replicated studies across different environmental gradients could be performed. The LD between the two SNP markers on chromosome 2 at ~7.543 and ~ 7.787 is 0.6711. The LD across the entire region from ~4.56 to ~16.27 Mb on chromosome 2 varies (Fig. 3). It seems like temperature‐based variables are shaping local adaptation much more strongly then precipitation‐based variables, especially precipitation accumulated over the growing season does not seem to cause strong patterns of local adaptation, at least in these stands.

The coefficients of determination of the linear regression between SNP genotypes and minimum daily temperature in October on chromosome 2 at ~7.543 Mb and at ~7.787 Mb are 0.3468 and 0.1646, respectively (Fig. 5). For the SNP maker at ~7.543 Mb, the homozygous G/G genotype only occurs in the high‐elevation population at Ruia, while the heterozygous genotype C/G is mostly restricted to the high elevation populations of Ruia and Lupului. On the other hand, the C/C genotype is present in similar frequencies in all populations. This pattern suggests strong environmental selection. This trend is not as pronounced for the marker at ~7.787 Mb on chromosome 2 (Fig. 5).

In a genome‐wide association study (GWAS) conducted on the same genotyping dataset as in this study, we observed significant associations between 88 SNPs located on chromosome 10 and stomatal density, as shown in Tost *et al*. (2025). These 101 markers are located on chromosome 10 in a region spreading from ~4.9 to 13.67 Mb, and also appear as one region (Tost *et al*. 2025). Five genes located in this region may play a role in controlling stomatal density (Tost *et al*. 2025). All markers within this region exhibit similar allele frequencies, which are correlated with stomatal density and the altitudinal gradient of the stands (see Tost *et al*. 2025). For the trait carbon isotope composition δ^13^C measured in 2020 and 2022, we also observed a peak on chromosome 10, which was much smaller and only resulted in 2020 in two significant SNP marker associations (Tost *et al*. 2025). Other significant markers associated with leaf nitrogen content or C/N ratio were also located on chromosome 2, but at ~30.26 Mb and ~31.804 Mb, ~14 Mb downstream of the region in this study (Tost *et al*. 2025). For the significant SNP marker on chromosome 2 at ~31.804 Mb associated with C/N ratio measured in 2021, the gene variant *Bhaga_2.g3457* was found based on a literature review. *Bhaga_2.g3457* was annotated as *abscisic‐aldehyde oxidase‐like* (National Center for Biotechnology Information (NCBI) 2025). The gene *abscisic‐aldehyde oxidase‐like* is responsible for oxidation of abscisic aldehyde, which is the last step of abscisic acid (ABA) biosynthesis (Seo *et al*. 2000). Popko *et al*. (2010) under drought stress observed auxin and abscisic acid (ABA) interact to regulate plant water status in poplar. The markers identified in our EAA and the GWAS do not overlap, possibly because of insufficient power of the analyses and multiple traits under environmental selection.

### Permutation testing

For the significance thresholds based on the BH correction, an average of 39 false‐positive results were observed for the 10 different permutations with 10 replications (Table S6). We identified 446 SNP markers significantly associated with the first principal component (PC), which are putatively true positive observations. Based on our permutation tests, we calculated a false positive rate of ~0.09. This false‐positive rate of ~0.09 is relatively low, so we did not adjust the significance thresholds. However, five permutation tests returned relatively high numbers of false positive observations (Table S6). These high numbers of false positives could be explained by partly still remaining elevational gradient structure. However, five permutations show very low numbers of false positive observations (Table S6). In conclusion, we can assume that spurious associations related to not fully separated signals of selection from those of demographic history exist, but are relatively small.

## CONCLUSIONS

In conclusion, our research confirms that environmental association analysis (EAA) can identify environmental variables, which contribute to local adaptation. We observed that mainly temperature‐based variables were detected with EAA and showed the strongest correlation with minor allele frequency (MAF) at significant SNP markers from the EAA. Consequently, the adaptive potential to temperature along a steep environmental gradient in the European beech populations was relatively high. A region for local adaptation on chromosome 2 from ~4.56 to ~16.27 Mb was identified based on this study and associated with all environmental variables except precipitation accumulated over the growing season. It seems like temperature‐based variables shaped local adaptation. The candidate SNP markers on chromosome 2 at ~7.543 (*Bhaga_2.g857*) and at ~7.787 (*Bhaga_2.g883*) were annotated with the genes *polygalacturonase QRT3‐like* and *NRT1/PTR_FAMILY 5.4‐like*. The gene *polygalacturonase QRT3‐like* plays a role in pollen development in *Arabidopsis* and may be involved in bud burst in European beech. The gene *NRT1/PTR_FAMILY 5.4‐like* belongs to the NRT1/PTR family, which is involved in the transportation of phytohormones like auxin. The MAF observed at these candidate SNP markers is increased, especially in Ruia, the population at highest altitude. Both candidate SNP markers (in *polygalacturonase QRT3‐like* and *NRT1/PTR_FAMILY 5.4‐like*) are annotated at or close to a part of the coding region where a codon change replaces a hydrophilic with a hydrophobic amino acid. These codon changes may increase the stability of proteins involved in auxin transport and reproductive processes such as pollen development. Assumptions about the function of these genes are speculative, because, in order to substantiate these assumptions, expression levels of the genes need to be measured under different temperature and watering regimes. Future research should also include replicates of environmental gradients.

## AUTHOR CONTRIBUTIONS

MT wrote the the initial draft, reviewed and edited the manuscript under supervision of OG. OGZ and ALC conducted the research, investigation process and managed the activities to sample the data. The analysis was conducted by MT and SW. The visualization of the data was done by MT. OG, ALC and HW planned and conceptualized the study. OG and HW acquired financial support for the project and oversaw the pro‐ject administration. OG, ALC, HW and MM developed the design and methodology used in this study.

## FUNDING INFORMATION

The used data come from the drought markers project (Reference numbers: 2218WK43B4, 2218WK43A4). This work was financially supported by the Federal Ministry of Food and Agriculture – FNR‐Waldklimafonds. The drought markers project is a collaboration between the University of Goettingen (Germany), the HAWK (Germany), the NW‐FVA (Germany) and Transylvania University of Brasov (Romania). We utilized the computational resources of the University of Goettingen's GWDG. Open Access funding enabled and organized by Projekt DEAL.

## CONFLICT OF INTEREST STATEMENT

The authors declare that there is no conflict of interest.

## Supporting information


**Fig. S1.** Distribution of diameter at breast height (DBH) measurements across stands.
**Fig. S2.** Distribution of environmental variables across stands with the precipitation based variables CHE_pr_12, CHE_pr_11, CHE_pr_10, CHE_pr_09, CHE_pr_08, CHE_pr_07, CHE_pr_06, CHE_pr_05, CHE_pr_04, CHE_pr_03, CHE_pr_02 and CHE_pr_01 (precipitation in December, November, October, September, August, July, June, May, April, March, February and January) and temperature‐based variables tas12, tas11, tas10, tas09 tas08, tas07, tas06, tas05, tas04, tas03, tas02 and tas01 (temperature in December, November, October, September, August, July, June, May, April, March, February and January).
**Fig. S3.** Correlations of all environmental variables and their correlation coefficients. All correlations are significant at *P* ~ 0***.
**Fig. S4.** Principal component analysis (PCA) based on all 53 environmental variables with principal component 1 plotted against principal component 2 (A) and the eigenvalues of the other principal components.
**Fig. S5.** Distribution of missingness (marker coverage) across samples before lfmm imputation with cut‐off threshold at a missingness of 0.05 (red) which corresponds to a marker coverage of 0.95.
**Table S6.** Randomization scheme and results from permutations (perm.) with number of false positive observations per replication. In the randomization scheme the order of the stands Ruia (Ru), Lupului (Lu), Solomon (So), Tampa (Ta) and Lempes (Le) was changed. The table also shows the initial order of the stands and the results from the not permuted analysis.
**Table S7.** Analysis of molecular variance (AMOVA) to assess the variation between stands and within stands with the poppr R package (Kamvar *et al*. 2015) expressed in %.
**Table S8.** Pairwise F_ST_ matrix calculated based on all SNP markers with the StAMPP R package (Pembleton *et al*. 2013).
**Fig. S9.** Manhattan plots based on LFMM results for environmental PC 2 (a), PC 3 (b), PC 4 (c), PC 5 (d), PC 6 (e), PC 7 (f), PC 8 (g) with Benjamini‐Hochberg (BH) corrected *P*‐values on the –log10.
**Fig. S10.** Manhattan plots based on LFMM results for mean precipitation (a), daily mean temperature (b), daily maximum (c), daily minimum temperature in July (d), elevation (e), EQ (f) and precipitation accumulated over the growing season (gsp) (f) with Benjamini‐Hochberg (BH) corrected *P*‐values on the –log10.
**Fig. S11.** Correlation between with different environmental variables tas01, tas06, tas09 (maximum daily temperature in January, June, September) and tasMAX01, tasMAX06, tasMAX09 (daily temperature in January, June, September) at the significant marker on chromosome 2 at ~7.543 (*Bhaga_2.g857*) and at ~7.787 (*Bhaga_2.g883*). These markers were annotated with gene variant *Bhaga_2.g857*, with the underlying gene *polygalacturonase QRT3‐like* and with gene variant *Bhaga_2.g883*, with the underlying gene protein *NRT1/ PTR FAMILY 5.4‐like*.
**Table S12.** Minor allele frequency (MAF) observed in the different stands at the significant markers (*P* ≤ 0.0001) on chromosome 2 associated with the different environmental variables tasMAX01, tasMAX02, tasMAX06, tasMAX09, tasMAX10, tasMAX11, tasMAX12 (maximum daily temperature in January, February, June, September, October, November and December), tasmin01, tasmin02, tasmin07, tasmin11, tasmin12 (minimum daily temperature in January, February, July, November, December) and elevation and their mean.
**Fig. S13.** Correlation between tasmin10 (minimum daily temperature in October) and the minor allele frequency (MAF) of the significant marker (*P* ≤ 0.0001) on chromosome 2 at (a) ~10.07112 Mb at‐0.8566 (*P*‐value = 0.0638), (b) ~10.07115 Mb at −0.8481 (*P*‐value = 0.0694), (c) ~10.526 Mb at −0.9201 (*P*‐value = 0.0268), (d) ~10.533 Mb at −0.9119 (*P*‐value = 0.0309), (e) ~11.929 Mb at −0.9055 (*P*‐value = 0.0344), (f) ~11.989 Mb at −0.9012 (*P*‐value = 0.0367), (g) ~14.682 Mb at −0.9236 (*P*‐value = 0.0250), (h) ~14.823 Mb at −0.8929 (*P*‐value = 0.0414), (i) ~15.895 Mb at −0.9148 (*P*‐value = 0.0295), (j) ~16.130 Mb at −0.8745 (*P*‐value = 0.0523).
**Table S14.** List of gene descriptions close to markers associated with environmental PC1.
**Fig. S15.** Coding region of the gene variant *Bhaga_2.g857* (*polygalacturonase QRT3‐like*) and the significant marker on chromosome 2 at ~7.543 Mb (green).
**Table S16.** Observed codon changes which lead to an amino acid (AA) replacement and a resulting change in polar bonds (hydrophobic or hydrophilic) at the underlying gene variants of markers associated with stomatal density.
**Fig. S17.** Coding region of the gene variant *Bhaga_2.g883* (*NRT1/PTR_FAMILY 5.4‐like*) and the significant marker on chromosome 2 at ~7.787 Mb (green).

## Data Availability

Raw data are publicly available at figshare: https://figshare.com/articles/dataset/VCF_file_with_SPET_sequencing_data_of_DroughtMarkers_project/28748924 and published by Tost *et al*. (2025). Our analysis code is available publicly on github: https://github.com/MilaTost/DroughtMarkers.
